# Association between Lifestyle and Gastroesophageal Reflux Disease Questionnaire Scores: A Cross-Sectional Study of 37 442 Chinese Adults

**DOI:** 10.1155/2019/5753813

**Published:** 2019-11-16

**Authors:** Yan Gong, Qiang Zeng, Yi Yan, Chaojing Han, Yansong Zheng

**Affiliations:** ^1^Health Management Institute, Chinese PLA General Hospital, Beijing 100853, China; ^2^Medical Director Quan Care Llc, 53 Elizabeth Street, Suite 4C, New York, NY 10013, USA

## Abstract

The aim of this study was to investigate the distribution characteristics of GerdQ results in a Chinese population and the association between lifestyle and GerdQ scores. Among the 37 442 individuals enrolled from September 2009 to March 2016, 7 449 (19.89%) had a GerdQ score of ≥8 points and 29 993 (80.11%) had a GerdQ score of <8 points. The percentage of men with suspected GERD was significantly higher than the percentage of women with suspected GERD (*χ*^2^ = 111.571, *P* ≤ 0.001), and the prevalence of GERD was higher in the young and middle-aged populations than in the elderly population. The prevalence of GERD increased gradually with weight gain (*χ*^2^ = 145.227, *P* ≤ 0.001). With regard to lifestyle, the prevalence of GERD in the subjects who smoked (*χ*^2^ = 119.361, *P* ≤ 0.001), consumed alcohol excessively, lacked physical activity (*χ*^2^ = 86.916, *P* ≤ 0.001), and had an excessive intake of oil, meat, fish, and eggs showed an ascending trend (*χ*^2^ = 105.388, *P* ≤ 0.001). In contrast, an adequate intake of vegetables (≥300 g/d) and fruit (≥200 g/d) was associated with a significantly lower incidence of GERD. Suspected GERD is very common in individuals undergoing health examinations. Unhealthy lifestyles are closely related to the high incidence of suspected GERD. GerdQ scores can play a role in screening for GERD.

## 1. Introduction

Gastroesophageal reflux disease (GERD) refers to the symptoms and complications caused by the backflow of gastric content into the oesophagus, mouth (including the throat), or lungs [1]. Reflux oesophagitis (RE) occurs when the oesophageal mucosa is damaged by acid (alkali) reflux. Endoscopy is a necessary means to diagnose oesophagitis. According to the results of endoscopic examination, GERD can be divided into nonerosive gastroesophageal reflux disease with negative endoscopic examination (NERD) and reflux oesophagitis with positive endoscopic examination. The complications of GERD may be the oesophageal mucosa break, ulcer or peptic stricture, Barrett's oesophagus (BE), and even oesophageal carcinoma [2]. The common symptoms of GERD include heartburn, regurgitation, nausea, belching, and angina-like retrosternal chest pain. However, many patients may present with atypical symptoms, such as abnormal sensation of the pharynx, chest tightness, shortness of breath, cough, and asthma, which often led to missed diagnosis and misdiagnosis [3].

At present, GERD is often diagnosed by the following five methods: (a) a questionnaire to learn the frequency and severity of symptoms; (b) upper gastrointestinal endoscopy; (c) proton pump inhibitor (PPI) treatment as a diagnostic test; (d) 24-h oesophageal pH monitoring; and (e) combined 24-h and impedance-pH monitoring [4]. Endoscopy and 24-h oesophageal pH monitoring results are regarded as the “gold” standard for the diagnosis of GERD. However, high cost, poor operability, and low patient compliance have limited their applications. In 2006, the disease GERD was defined as “troublesome symptoms and/or complications” resulting from gastroesophageal reflux [5], and the gastroesophageal reflux disease questionnaire (GerdQ) was then designed. GerdQ is a self-administered 6-item questionnaire that was recently developed as a tool to improve and standardize the symptom-based diagnosis and evaluation of treatment response in primary care patients with GERD [4]. Studies have shown that the GerdQ has a sensitivity of 65% and a specificity of 71% [5–7]. Chinese experts have also recognized its value in diagnosing GERD [8]. To the best of our knowledge, however, only a few studies with a large sample size of participants receiving health check-ups have described the application of GerdQ in screening GERD.

Many studies have focused on the association between GERD and established risk factors, such as age, gender, body mass index (BMI) [9], obesity [10], tobacco smoking [11], and physical activity [12]. The results indicated that mild routine physical activity in association with diet modifications, i.e., a diet rich in fibre and low in fat, seemed to be advisable to prevent reflux symptoms. Recently, Mone et al. highlighted a beneficial effect of a Mediterranean diet in the occurrence of GERD [13]. However, only one study from Japan evaluated lifestyle factors affecting gastroesophageal reflux disease symptoms in a healthy checkups group using scores on the frequency scale for the symptoms of GERD (FSSG). The FSSG is a widely used questionnaire for the diagnosis of GERD and for evaluating the effectiveness of the treatment. There are twelve questions of the FSSG that cover various symptoms related to the upper gastrointestinal tract as well as psychosomatic symptoms; a score of more than seven points suggested the presence of GERD in the respondent [14]. Very few large-scale population-based studies have been performed on lifestyle and GerdQ scores.

Therefore, in the present study, we first analysed the incidence of GERD. Second, we explored the potential association between daily lifestyles and the incidence of GERD in an attempt to further investigate the application of the GerdQ in diagnosing GERD in physical examination populations.

## 2. Materials and Methods

### 2.1. Study Population

Individuals who had undergone a routine physical examination in the Health Medical Center of the PLA General Hospital from September 2009 to March 2016 were enrolled as the subjects of this study. Patients with stroke, heart failure, renal insufficiency, malignant tumour, and/or gastric surgery were excluded. Individuals with incomplete examination and test records were also excluded. For patients who had received multiple physical examinations, the results of the latest physical health checkup were used. A total of 37 442 subjects were enrolled, including 27 274 men (72.84%) and 10 168 women (27.16%).

### 2.2. Sampling and Subject Recruitment

The survey content included general demographic information, smoking and drinking histories, diet and exercise habits, and physical examination and laboratory findings. Physical examinations included the measurement of height, weight, and blood pressure, whereas laboratory tests included routine items such as blood lipids and blood glucose. Smoking is defined as consuming ≥10 cigarettes per day for at least 1 year, and smoking cessation is defined as the continuous quitting of smoking for more than one year, which was consistent with the World Health Organization (WHO) definition for smoking [15].

Alcohol consumption is classified as limited alcohol consumption (never drinking or alcohol drinking not exceeding 25 g/d in men or not exceeding 15 g/d in women) and excessive alcohol consumption (average daily alcohol consumption exceeds 25 g in men or exceeds 15 g in women). It was based on the WHO Global Status Report on Alcohol and Health 2014 and the Dietary Guidelines for Chinese Residents 2016 [16, 17].

Blood pressure was measured in accordance with the Chinese Hypertension Prevention and Treatment Guidelines 2005, which is defined as a systolic blood pressure of ≥140 mmHg and/or diastolic blood pressure of ≥90 mmHg [18].

A body composition analyser was used to measure body weight and height, and the BMI was calculated as weight (kg)/height^2^ (m^2^), according to WHO standards [19]. Values of overweight are defined as a body mass index (BMI) of 24 kg/m^2^ to 28 kg/m^2^ and obesity as a BMI of ≥28 kg/m^2^, according to the 2016 “Expert consensus on medico-nutritional treatment of overweight and obesity in China” [20].

All subjects fasted overnight and wore a hospital gown during the measurements. This study was approved by the Ethics Committee of the PLA General Hospital, Beijing, China. All subjects were informed that their health checkup data might be used anonymously in scientific research and signed informed consent.

### 2.3. GerdQ and Questionnaire

During the GerdQ-based survey, the subjects were asked to recall their symptoms and the frequency of their occurrence over the past 7 days. The symptom frequencies were scored as follows: (a) the frequency of positive symptoms (heartburn and reflux): 0, 1, 2, and 3 points for 0 day, 1 day, 2-3 days, and 4-7 days, respectively, with the highest possible score being 6 points; (b) the frequency of negative symptoms (upper abdominal pain and nausea): 3, 2, 1, and 0 points for 0 day, 1 day, 2-3 days, and 4-7 days, respectively, with the highest possible score being 6 points; and (c) the frequency of heartburn and reflux affecting sleep at night and the need for additional medications: 0, 1, 2, and 3 points for 0 day, 1 day, 2-3 days, and 4-7 days, respectively. The sum of the points for the abovementioned frequencies served as a subject's GerdQ scores, and a diagnosis of GERD could be made if the sum was ≥8 points [4].

The judgement criteria for dietary factors were as follows: (a) standard daily intake of cereals (including coarse cereals): 250 g-400 g; nonstandard if <250 g or >400 g; (b) standard daily vegetable (≥300 g) and fruit (≥200 g) consumption; (c) standard daily intake of fish and shrimp (50 g-100 g), livestock and poultry meat (50 g-75 g), and eggs (25 g-75 g); inadequate if <125 g and excessive if >225 g; (d) standard daily edible oil consumption: <30 g; (e) standard alcohol consumption: ≤25 g for men and ≤15 g for women or never drinking; and (f) standard daily intake of dairy products: 250 g-350 g; inadequate if <250 g and excessive if >350 g. These classifications are derived from the general recommendations of the WHO panel on diet, nutrition, and chronic disease prevention and the Dietary Guidelines for Chinese Residents 2016 [17, 21, 22].

The judgement criteria for physical activity were as follows: (a) adequate physical activity: engaging in vigorous activity for at least 3 days per week (with a minimum total physical activity of 1500 metabolic equivalent- (MET-) min per week) or 7 days a week (achieving a total physical activity of at least 3000 MET-min per week); (b) moderate physical activity: engaging in at least 20 minutes of vigorous physical activity per day for a minimum of 3 days per week or at least 30 minutes of nonvigorous physical activity per day for a minimum of 5 days per week, or at least 5 days of physical activity per week, achieving a minimum of 600 MET-min per week but not exceeding 3000 MET-min; (c) inadequate physical activity: if the abovementioned criteria (which meet WHO recommendations on physical activity for health) are not met [23].

### 2.4. Statistical Analysis

The survey data were encoded, quantified, and entered into a computer. Statistical analysis was performed using Stata 11.0 software (http://www.stata.com/stata11/). The normality was tested by the Kolmogorov-Smirnov method. The measurement data are expressed as the means ± standard deviations, and the categorical data are expressed as percentages. Chi-squared tests and logistic regression analyses were performed, and a *P* value of <0.05 was considered statistically significant.

## 3. Results

### 3.1. GerdQ-Based Survey

Among the 37 442 subjects enrolled in this study, 7 449 (19.89%) had a GerdQ score of ≥8 points and 29 993 (80.11%) had a GerdQ score of <8 points.

### 3.2. Comparisons of Clinical Data

**Figure 1:**
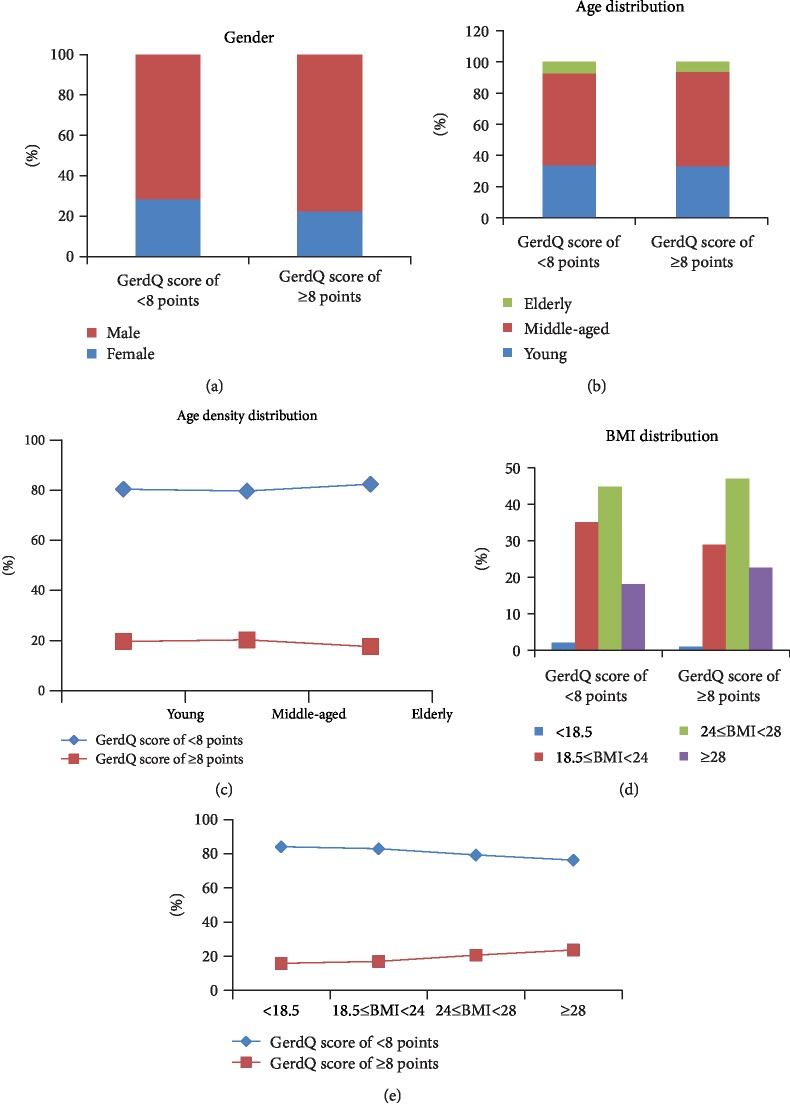
Distributions of clinical traits between the groups with GerdQ score of ≥8 points and GerdQ score of <8 points. (a) Sex distribution in these two groups. *Y*-axis represents sex composition. Blue color represents female individuals, and red color represents male individuals. (b) Age distribution of these two groups. (c) Different age groups' change curve of GerdQ score of ≥8 points and GerdQ score of <8 points. (d) Frequency of BMI among these two population groups. (e) Different BMI groups' change curve of GerdQ score of ≥8 points and GerdQ score of <8 points.

According to the GerdQ scores, the subjects were divided into two groups: the suspected GERD group and the non-GERD group. The results showed that the percentage of men with suspected GERD was significantly higher than the percentage of women with suspected GERD (*χ*^2^ = 111.571, *P* ≤ 0.001) (Figure 1(a)). The age distribution of the suspected GERD group and the non-GERD group was similar (Figure 1(b)). The difference in the prevalence of GERD was not significant between young and middle-aged subjects (*χ*^2^ = 2.342, *P* = 0.126) but was significantly higher in young and middle-aged subjects than in elderly subjects (*χ*^2^ = 12.042, *P* = 0.002), which shows a positive association between GERD and younger age (Figure 1(c)). The body weight distribution of these two groups was similar, and the prevalence of GERD was higher among overweight people (Figure 1(d)). The prevalence of GERD gradually increases with weight gain from low body weight, normal body weight, and overweight to obesity (*χ*^2^ = 145.227, *P* ≤ 0.001) (Figure 1(e)). Association analysis revealed stronger associations between the prevalence of GERD and body weight gain. Furthermore, waist circumstance, systolic blood pressure, diastolic blood pressure, and levels of triglycerides, cholesterol, and uric acid were significantly higher in the suspected GERD group than in the non-GERD group (*P* ≤ 0.001). Fasting blood glucose (*P* = 0.039) and LDL-C were also slightly higher (*P* = 0.002), while HDL-C was significantly lower (*P* ≤ 0.001) (Table 1).

### 3.3. Comparisons of Lifestyle

**Figure 2:**
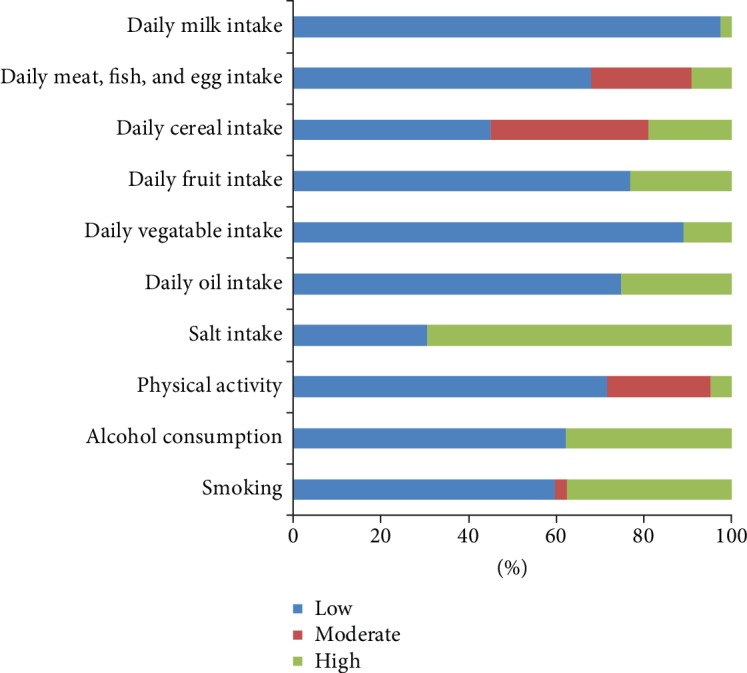
The correlation of ten background factors with the group of GerdQ score of ≥8 points. Blue color represents low-level/inadequate intake, red color represents moderate level intake, and green color represents high level/excessive/adequate intake.

The prevalence of GERD was highest in the smoking cessation group, followed by the smoking group, and the nonsmoking group (*χ*^2^ = 119.361, *P* ≤ 0.001). The proportion of suspected GERD was significantly higher in the excessive alcohol consumption group than in the nonexcessive alcohol consumption group. The rate of suspected GERD was the lowest in subjects with adequate physical activity, followed by subjects with moderate and inadequate levels of physical activity (*χ*^2^ = 86.916, *P* ≤ 0.001). In terms of dietary factors, subjects with excessive daily oil intake had a significantly higher incidence of GERD than did those with standard oil intake. The prevalence of GERD was significantly lower in subjects with standard vegetable (≥300 g/day) and fruit (≥200 g/day) intake than in those who did not meet the standards. The intake of dairy products showed no significant effect on GERD incidence. The prevalence of GERD was not significantly different between the low-cereal-intake group and the moderate-cereal-intake group (*P* = 0.317) but was significantly lower than in the high-cereal-intake group. The prevalence of GERD gradually increased in subjects with inadequate, adequate, and excessive consumption of meat, fish, and eggs (*χ*^2^ = 105.388, *P* ≤ 0.001) (Figure 2, Table 2).

### 3.4. Multivariate Analysis of Suspected GERD

Based on the results of univariate analysis, a stepwise multivariate regression analysis was performed with suspected GERD as the dependent variable and the following as independent variables: gender; smoking; alcohol consumption; salt intake; physical activity; age stratification; BMI stratification; waist circumference; systolic blood pressure; diastolic blood pressure; TC; TG; uric acid; fasting blood glucose; LDL-C; HDL-C; and daily intake of oil, dairy products, vegetables, fruits, cereals, milk, and meat/fish/eggs. The results are shown in Table 3. Smoking (OR = 1.190, 95%CI = 1.120‐1.264), alcohol consumption (OR = 1.278, 95%CI = 1.207‐1.353), salt intake (OR = 0.903, 95%CI = 0.853‐0.956), physical activity (OR = 0.846, 95%CI = 0.808‐0.886), age stratification (OR = 1.080, 95%CI = 1.032‐1.132), waist circumference (OR = 1.014, 95%CI = 1.011‐1.017), TG (OR = 1.039, 95%CI = 1.021‐1.057), fasting blood glucose (OR = 0.966, 95%CI = 0.948‐0.985), and daily intake of fruits (OR = 0.910, 95%CI = 0.856‐0.967) and meat/fish/egg (OR = 1.088, 95%CI = 1.042‐1.135) were significantly associated with the prevalence of suspected GERD.

## 4. Discussion

This was the first large-scale study exploring the prevalence of GERD-related symptoms using GerdQ scores in a sample of individuals receiving health checkups in China. Our data showed a prevalence rate of weekly symptoms in 19.89% of the total subjects. The GerdQ is a simple, rapid, noninvasive, and effective tool that can reliably reflect changes in symptoms and quality of life. It is easy to operate, with minor memory bias and good patient compliance; furthermore, it has a sensitivity of 60%-90% with a specificity of 45%-86%, which is the diagnostic accuracy of a gastroenterologist [4, 6]. Therefore, the GerdQ is a good screening tool for the preliminary diagnosis of GERD [7].

The prevalence of GERD is geographically and racially related in GERD epidemiology [24–26]. The incidence of GERD has been estimated to range from 18.1% to 27.8% in North America, 8.8% to 25.9% in Europe, and 2.5% to 7.8% in East Asia [24]. In a previous study performed in China, 3 338 people (response rate 95.0%) aged 18–90 years randomly selected from southern China were enrolled in a survey [27]. The results showed that the prevalence of weekly heartburn and regurgitation was 2.2% and 7.0%, respectively, and the prevalence of weekly heartburn and/or regurgitation was 7.8%. In another report from China, a survey was conducted in 2007 among 16 078 individuals (response rate 89.3%) aged 18–80 years randomly sampled from urban and rural areas of Shanghai, Beijing, Wuhan, Xi'an, and Guangzhou. Overall, the prevalence of weekly heartburn and/or regurgitation was 5.2%, varying among different regions from 3.2% to 7.5% [28]. Our data implied a prevalence rate of weekly symptoms in 19.89% of the total respondents (7 449/37 424). With changes in lifestyles and the Westernization of diets, the prevalence of GERD is increasing in China; the results of our study were consistent with the values reported in the literature.

In the present study, the proportion of suspected GERD was significantly higher in men than in women (*χ*^2^ = 111.571, *P* ≤ 0.001), and the proportion of GERD was high in younger and middle-aged subjects.

Differences in the proportion of suspected GERD between men and women may have emerged simply because the population screened was ~70% men and ~30% women. It is worthwhile to investigate the percentage of men and women who had suspected GERD.

Further dietary analysis suggested that excessive alcohol consumption, excessive daily salt and fat intake, inadequate intake of vegetables and fruits, excessive intake of cereals, and excessive intake of meat, fish, and eggs were associated with a high incidence of GERD. It has been reported that the incidence of GERD was high among the residents in north-western China, who have a preferred diet of beef and mutton [29]. Similarly, individuals who consume more coffee, tea, and/or citrus fruits and are more prone to GERD [30]. Several studies found that fruits, vegetables, and high-fibre diets are inversely associated with GERD [31], whereas a recent monozygotic twin-based epidemiological study in Sweden showed that none of these items was associated with the risk of GERD symptoms [32]. In conclusion, the role of diet, especially specific foods or drinks, in GERD clinical symptoms warrants further study, although some dietary interventions continue to be recommended as first-line therapy for GERD relief. Larger prospective controlled trials are required to conclusively modify recommended dietary guidelines in the treatment of GERD.

Recent studies have also shown that smoking is an independent factor in predicting the progression of GERD [33, 34]. However, smoking cessation was associated with improved GERD symptoms only in individuals with normal BMI (not including overweight populations) [11]. Obesity and unhealthy dietary habits may increase the risk of GERD via weight gain [35], suggesting that high BMI, which is an indicator of general obesity, is one of the risk factors that can be used to predict the progression and severity of GERD symptoms [36, 37]. Central obesity, based on waist circumstance, is an independent factor associated with GERD. Excessive abdominal fat can increase the risk of GERD by increasing intragastric pressure and lowering lower oesophageal sphincter (LES) pressure and transient lower oesophageal sphincter relaxation (TLESR), thus leading to acid reflux [38]. In the present study, the prevalence of GERD was high in populations with inadequate physical activity, overweight, or obesity. In addition, subjects with suspected GERD had higher waist circumference, blood pressure, blood glucose, TC, TG, LDL-C, and uric acid levels than in non-GERD subjects, but HDL-C was lower. Thus, lifestyle modification has been increasingly recognized as one of the most important means of treating GERD, as supported by an increasing number of studies [39–41].

The present study had several strengths. It was based on a large sample size, and the results were highly representative of the Chinese population. All questionnaires were completed by the subjects themselves, and the data acquired were highly reliable. We found that 19.89% of the subjects receiving health checkups had a GerdQ score of ≥8 points. Although GERD cannot be diagnosed by GerdQ scores alone, this method is cost-effective and of great practical significance in preliminary screenings for GERD in individuals receiving health checkups to identify suspicious or risky cases and offer appropriate lifestyle interventions.

Our study had several limitations. First, the GerdQ provides a symptom-based diagnosis; some patients with GERD may present with atypical symptoms, such as cough, asthma, laryngitis, and the absence of typical oesophageal symptoms, while other patients with GERD experience no symptoms at all [1, 38]. In addition, heartburn and regurgitation may be related to other digestive diseases, such as functional dyspepsia. As a consequence, our study described a suspected GERD group according to GerdQ scores. However, such limitations represent a common problem for all population-based studies aimed at investigating the prevalence of GERD through questionnaires. The second limitation of our study is that this is a single-centre study of Chinese adults; thus, these results cannot be generalized to other racial and ethnic groups.

## 5. Conclusion

In conclusion, suspected GERD or a high risk of GERD is very common in the population of individuals undergoing health examinations. Unhealthy lifestyles are closely related to a high incidence of suspected GERD. GerdQ scores can play a role in screening for GERD. Larger prospective controlled trials are required to conclusively modify the recommended dietary guidelines in the treatment of GERD.

## Figures and Tables

**Table 1:** Comparisons the clinical data between GerdQ score of ≥8 points and GerdQ score of <8 points.

Features	GerdQ score of <8 points (*n* = 29 993)	GerdQ score of ≥8 points (*n* = 7 449)	*χ* ^2^ value	*t* value	*P* value
Gender			111.571		≤0.001^∗^
Female	8 508 (83.67%)	1 660 (16.33%)			
Male	21485 (78.77%)	5789 (21.23%)			
Age			12.042		0.002^∗^
Young	10 060 (80.38%)	2 456 (19.62%)			
Middle-aged	17 710 (79.67%)	4 518 (20.33%)			
Elderly	2 223 (82.39%)	475 (17.61%)			
BMI			145.227		≤0.001^∗^
<18.5	579 (84.16%)	109 (15.84%)			
18.5 ≤ BMI<24	10 535 (83.02%)	2 154 (16.98%)			
24 ≤ BMI< 28	13 443 (79.35%)	3 499 (20.65%)			
≥28	5 436 (76.32%)	1 687 (23.68%)			
Waist circumstance	87.99 ± 10.19	90.09 ± 10.08		16.004	≤0.001^∗^
Systolic blood pressure	122.06 ± 15.24	122.99 ± 14.89		4.760	≤0.001^∗^
Diastolic blood pressure	81.93 ± 11.43	83.13 ± 11.22		8.107	≤0.001^∗^
Fasting blood glucose (mmol/L))	5.88 ± 1.45	5.91 ± 1.45		2.059	0.039^∗^
TC (mmol/L)	4.84 ± 0.95	4.89 ± 0.94		4.245	≤0.001^∗^
TG (mmol/L)	1.79 ± 1.43	1.98 ± 1.57		9.966	≤0.001^∗^
HDL-C (mmol/L)	1.28 ± 0.33	1.24 ± 0.32		9.137	≤0.001^∗^
LDL-C (mmol/L)	3.11 ± 0.82	3.14 ± 0.82		3.031	0.002^∗^
UA (*μ*mol/L)	340.52 ± 87.41	350.51 ± 87.26		8.812	≤0.001^∗^

BMI: body mass index; TC: cholesterol; TG: triglyceride; HDL-C: high-density lipoprotein cholesterol; LDL-C: low-density lipoprotein cholesterol; UA: uric acid. Univariate regression analysis was performed focusing on the 12 background factors, comprised of two continuous variables (age and BMI) and other 10 categorical variables. Student's *t* test was used to evaluate the correlation between each background factor and GerdQ score (mean ± standard deviation is shown). The level of significance in each factor was set at *P* value <0.05 (^∗^).

**Table 2:** Comparisons of the lifestyles between GerdQ score of ≥8 points and GerdQ score of <8 points.

Features	GerdQ score of <8 points (*n* = 29 993)	GerdQ score of ≥8 points (*n* = 7 449)	*χ* ^2^ value	*P* value
Smoking			119.361	≤0.001
Non-smoking	19 909 (81.76%)	4 443 (18.24%)		
Smoking cessation	657 (76.22%)	205 (23.78%)		
Smoking	9 427 (77.09%)	2 801 (22.91%)		
Alcohol consumption			211.635	≤0.001
Nonexcessive	21 237 (82.11%)	4 626 (17.89%)		
Excessive	8 756 (75.62%)	2 823 (24.38%)		
Physical activity			86.916	≤0.001
Inadequate	19 827 (78.78%)	5 339 (21.22%)		
Moderate	8 305 (82.52%)	1 759 (17.48%)		
Adequate	1 861 (84.13%)	351 (15.87%)		
Salt intake			80.598	≤0.001
Control intake	10 856 (82.62%)	2 283 (17.38%)		
Over intake	19 137 (78.74%)	5 166 (21.26%)		
Daily oil intake			91.419	≤0.001
≥35 g/d	9 263 (83.14%)	1 879 (16.86%)		
<35 g/d	20 730 (78.82%)	5 570 (21.18%)		
Daily vegetable intake			10.7289	0.001
≥300 g/d	3 704 (81.93%)	817 (18.07%)		
<300 g/d	26 289 (79.85%)	6 632 (20.15%)		
Daily fruit intake			45.222	≤0.001
≥200 g/d	8 111 (82.43%)	1 729 (17.57%)		
<200 g/d	21 882 (79.28%)	5 720 (20.72%)		
Daily cereal intake			6.465	0.039
<200 g/d	13 490 (80.13%)	3 346 (19.87%)		
≥200 g/d, <400 g	11 155 (80.58%)	2 688 (19.42%)		
≥400 g/d	5 348 (79.08%)	1 415 (20.92%)		
Daily meat, fish, and egg intake			105.388	≤0.001
<125 g/d	22 107 (81.36%)	5 066 (18.64%)		
≥125 g/d, <225 g/d	5 873 (77.46%)	1 709 (22.54%)		
≥225 g	2 013 (74.92%)	674 (25.08%)		
Daily milk intake			2.249	0.134
≥250 g/d	632 (78.02%)	178 (21.98%)		
<250 g/d	29 361 (80.15%)	7 271 (19.85%)		

Univariate regression analysis was performed focusing on the 10 background factors. The correlation of GerdQ score with 10 variables was assessed using chi-squared test and logistic regression analysis. The level of significance in each factor was set at *P* value <0.05 (^∗^).

**Table 3:** A stepwise multivariate regression analysis of suspected GERD.

Features	Odds ratio	Std. err.	*Z*	*P* > ∣*z*∣	[95% conf. interval]
Smoking	1.190	0.037	5.66	0.000	[1.120-1.264]
Alcohol consumption	1.278	0.037	8.40	0.000	[1.207-1.353]
Salt intake	0.903	0.026	-3.52	0.000	[0.853-0.956]
Physical activity	0.846	0.020	-7.14	0.000	[0.808-0.886]
Age stratification	1.080	0.025	3.28	0.000	[1.032-1.132]
Waist circumstance	1.014	0.001	9.62	0.000	[1.011-1.017]
TG (mmol/L)	1.039	0.009	4.25	0.000	[1.021-1.057]
Fasting blood glucose (mmol/L))	0.966	0.009	-3.51	0.000	[0.948-0.985]
Daily fruit intake	0.910	0.028	-3.04	0.000	[0.856-0.967]
Daily meat, fish, and egg intake	1.088	0.024	3.85	0.000	[1.042-1.135]

Multivariate regression analysis was performed focusing on the 10 background factors; the normality was tested by the Kolmogorov-Smirnov method. The correlation of GerdQ score with 10 variables was assessed using chi-squared test and logistic regression analysis. The level of significance in each factor was set at *P* value <0.05 (^∗^).

## Data Availability

The datasets used and/or analysed during the current study are available from the corresponding author on reasonable request.
